# Unveiling Tim-3 immune checkpoint expression in hepatocellular carcinoma through abdominal contrast-enhanced CT habitat radiomics

**DOI:** 10.3389/fonc.2024.1456748

**Published:** 2024-11-08

**Authors:** Zhishen Tang, Wei Wang, Bo Gao, Xuyang Liu, Xiangyu Liu, Yingquan Zhuo, Jun Du, Fujun Ai, Xianwu Yang, Huajian Gu

**Affiliations:** ^1^ Department of Pediatric Surgery, Affiliated Hospital of Guizhou Medical University, Guiyang, China; ^2^ School of Clinical Medicine, Guizhou Medical University, Guiyang, China; ^3^ Department of Radiology, Affiliated Hospital of Guizhou Medical University, Guiyang, China; ^4^ Department of Pathology and Pathophysiology, Guizhou Medical University, Guiyang, China

**Keywords:** hepatocellular carcinoma, Tim-3 expression, habitat radiomics, immunotherapy, bioanalysis

## Abstract

**Introduction:**

Immune checkpoint inhibitors (ICIs) are important systemic therapeutic agents for hepatocellular carcinoma (HCC), among which T-cell immunoglobulin and mucin-domain containing protein 3 (Tim-3) is considered an emerging target for ICI therapy. This study aims to evaluate the prognostic value of Tim-3 expression and develop a predictive model for Tim-3 infiltration in HCC.

**Methods:**

We collected data from 424 HCC patients in The Cancer Genome Atlas (TCGA) and data from 102 pathologically confirmed HCC patients from our center for prognostic analysis. Multivariate Cox regression analyses were performed on both datasets to determine the prognostic significance of Tim-3 expression. In radiomics analysis, we used the K-means algorithm to cluster regions of interest in arterial phase enhancement and venous phase enhancement images from patients at our center. Radiomic features were extracted from three subregions as well as the entire tumor using pyradiomics. Five machine learning methods were employed to construct Habitat models based on habitat features and Rad models based on traditional radiomic features. The predictive performance of the models was compared using ROC curves, DCA curves, and calibration curves.

**Results:**

Multivariate Cox analyses from both our center and the TCGA database indicated that high Tim-3 expression is an independent risk factor for poor prognosis in HCC patients. Higher levels of Tim-3 expression were significantly associated with worse prognosis. Among the ten models evaluated, the Habitat model constructed using the LightGBM algorithm showed the best performance in predicting Tim-3 expression status (training set vs. test set AUC 0.866 vs. 0.824).

**Discussion:**

This study confirmed the importance of Tim-3 as a prognostic marker in HCC. The habitat radiomics model we developed effectively predicted intratumoral Tim-3 infiltration, providing valuable insights for the evaluation of ICI therapy in HCC patients.

## 1 Introduction

Hepatocellular carcinoma (HCC) is the third leading cause of cancer-related deaths worldwide, posing a significant health problem globally (1). Chronic damage caused by viral infections or alcohol is the primary cause of HCC development (2, 3). Currently, the lack of effective predictors for antitumor efficacy and therapeutic approaches often limits the prognosis of HCC patients (4). Immune checkpoint therapy (ICI) has been developed for various advanced solid tumors to block signals that inhibit T cell activation and to reactivate the body’s antitumor immune response (5). Similar to PD-1/PD-L1 and CTLA-4, T-cell immunoglobulin and mucin domain-3 (Tim-3) is also an important member of the immune checkpoint family. Immunotherapies targeting Tim-3 have been widely applied in various solid tumors, including triple-negative breast cancer (6), lung cancer (7), and glioblastoma (8). Tim-3 belongs to the TIM family and was initially identified as a co-inhibitory receptor on T-helper 1 cells that regulates type I immune responses (9, 10). Subsequent studies found that Tim-3 is expressed on various cell types, including NK cells (11), macrophages (12), and mast cells (13), and plays a regulatory role in the tumor microenvironment (TME). A pan-cancer analysis (14) showed that upregulated Tim-3 is a risk factor associated with the overall survival (OS) of various tumors, suggesting that Tim-3 has potential as a prognostic biomarker. Recent studies have found that the number of Tim-3 positive cells in HCC tissues is a negative prognostic factor affecting overall survival (15). Tim-3 expression on tumor-associated macrophages is associated with poor prognosis (12). Therefore, Tim-3 has been identified in studies as a potential prognostic biomarker for HCC (16). Multiple preclinical studies have shown that Tim-3 can enhance antitumor immunity and inhibit the progression of HCC through various methods. These methods include using neoantigen immunotherapy gel combined with Tim-3 blockade (17), co-delivering Tim-3 siRNA and sorafenib via nanoparticles (18), and blocking Tim-3 signaling to prevent CD8+ T cell apoptosis (19), thereby enhancing the anti-HCC effect. This indicates that Tim-3 not only has prognostic predictive value but also potential therapeutic predictive value.

Multimodal studies can identify new prognostic biomarkers to help select patients who are more suitable for ICI therapy. However, rapidly obtaining high-throughput molecular data from tumors remains a challenge (20). Radiomics, as a rapid and non-invasive technique, can extract and quantify high-throughput information from images (21) It has been widely applied in predicting the prognosis of HCC (22), drug treatment response (23), pathological classification and immune status (24), and has also been used to predict the expression of PD-1 (25) and CTLA-4 (26). Habitat analysis, a product of radiomics development, is a method that uses tumor images to further classify and quantitatively analyze subregions within the tumor tissue that have different metabolic characteristics (27). This method has been applied to studies of breast cancer (28) and prostate cancer (29), demonstrating its unique advantages in analyzing the tumor TME. In brain tumors, it has been used to predict prognosis and metabolic pathways (30, 31). For example, Niha et al. used habitat analysis technology to construct a prognostic model for glioblastoma and successfully identified 192 clinically significant differentially expressed genes in the high and low-risk groups (32). Habitat analysis has also been used to predict HCC recurrence (33), demonstrating excellent predictive performance. This study validates the feasibility of Tim-3 expression as a potential prognostic biomarker and compares the utility of traditional radiomics methods and habitat radiomics methods for non-invasive preoperative prediction of the immune checkpoint Tim-3 expression. Our findings provide valuable insights into the non-invasive prediction of tissue biomarkers using habitat radiomics.

## 2 Materials and methods

### 2.1 Bioinformatics analysis

We used the Aclbi platform (https://www.aclbi.com/) to evaluate the differential expression of Tim-3 between HCC and normal tissues, and downloaded and organized RNAseq data from the TCGA-LIHC project through the Cancer Genome Atlas (TCGA) data portal (https://portal.gdc.cancer.gov/). After screening, 424 data samples were retained for analysis. In the data preprocessing, we converted the Tim-3 expression data to transcripts per million format. Spearman correlation analysis was used to evaluate the impact of Tim-3 on the immune TME, and the linkET package was used to visualize the correlation of tumor immune cycle scores. Prognostic data referenced a Cell article (34), using the survival package to perform proportional hazards assumption tests and Cox regression analysis. In univariate analysis, variables with p<0.05 were included in the multivariate Cox regression model, and the results were visualized using the forestplot package. Finally, we used the Kaplan-Meier Plotter (https://kmplot.com/analysis/index.php?p=background) to analyze the relationship between Tim-3 expression and OS in the Asian population. All the analyses mentioned above were conducted using TCGA data.

### 2.2 Local dataset

This study adopted a retrospective design, with informed consent obtained from all participants. Approval was granted by the local ethics committee (Ethics Approval No.: 2021 LUN Review No.: 663), and all methods were conducted in accordance with relevant guidelines. We included 150 patients who underwent R0 hepatic resection in the Department of Hepatobiliary Surgery at our hospital from January 2015 to November 2018. The inclusion criteria for this study were:

1. Patients who underwent CECT of the upper abdomen within one month before surgery;
2. Patients who had not received any antitumor treatment before surgery; if postoperative CT indicated signs of recurrence, the patient received radiofrequency ablation therapy;
3. Patients with pathology reports confirming primary HCC.

Patients were excluded from this study if they met any of the following criteria:

1. Patients with missing or incomplete imaging data or clinical information;
2. Patients who did not reach the follow-up endpoint;
3. Patients with other types of malignancies;
4. Patients whose immunohistochemical results showed no malignant tumor cells.

**Figure 1:**
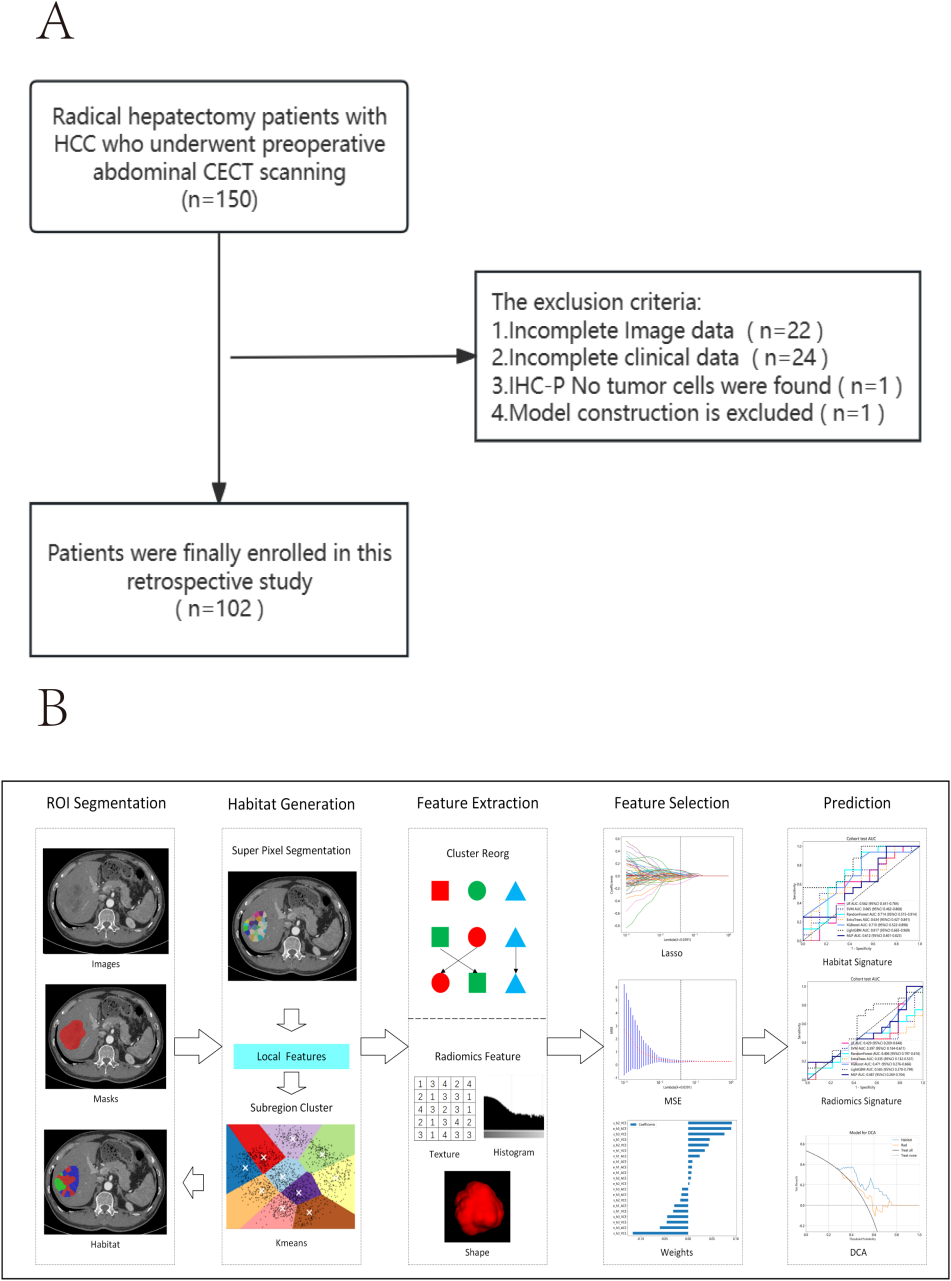
**(A)** Workflow of inclusion and exclusion criteria. **(B)** Habitat radiomics workflow.

The inclusion and exclusion criteria are illustrated in **Figure 1A**.

### 2.3 Histopathological evaluation of Tim-3 infiltration status in HCC

Paraffin-embedded tissue from surgical resection specimens was cut into 5 mm thick sections, deparaffinized, hydrated, and subjected to antigen retrieval. The sections were then incubated with a primary antibody against Tim-3 overnight. After incubation with a secondary antibody, the specimens were stained using DAB and counterstained with hematoxylin, followed by dehydration and mounting.

To quantify the infiltration level of Tim-3 positive cells in tumor samples, immunohistochemistry was performed three times for each patient, and one random field of view (400X) was selected. The staining intensity and extent were automatically scored using the IHC Profiler in ImageJ (v1.54). The final H-score was calculated using the following formula:


H−score=3×High Positive+2×Positive+1×Low Positive+0×Negative

**Figure 2:**
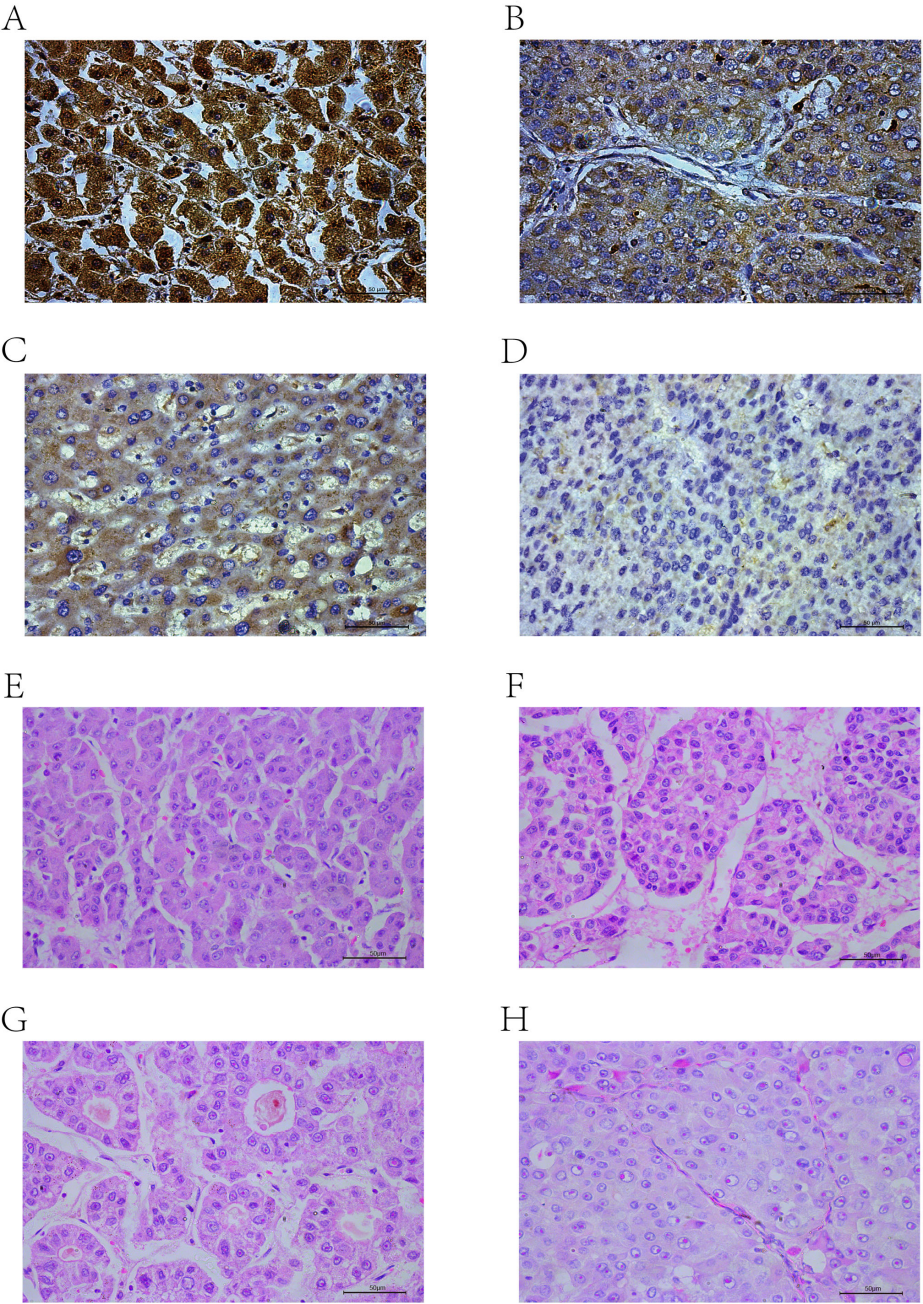
IHC-P results: **(A)** High positive. **(B)** Positive. **(C)** Low positive. **(D)** Negative. HE results: **(E)** Trabecular-micro. **(F)** Trabecular-macro. **(G)** Pseudoglandular. **(H)** Compact.

The scores ranged from 0 to 300, with 300 indicating that 100% of tumor cells in the field were strongly stained (**Figures 2A–D**). The expression levels were classified based on the optimal cut-off value for OS determined by X-tile software (35): low (H-score ≤ 92.3) or high (H-score > 92.3).

### 2.4 Histopathological evaluation of HCC subtypes using HE staining

We combined postoperative pathology reports and used hematoxylin and eosin (HE) staining to re-evaluate the histopathological subtypes of HCC. Tissue sections were deparaffinized, hydrated, and stained with hematoxylin and eosin, followed by dehydration and mounting. The slides were then examined under a microscope, with one random field of view (400X) selected for each patient. For patients with indeterminate pathology due to the presence of two subtypes, three random fields of view were selected to assess the proportion of each subtype. The predominant subtype was used to define mixed pathology patients.

The histological subtypes of HCC are generally classified into trabecular-micro (**Figure 2E**), trabecular-macro (**Figure 2F**), pseudoglandular (**Figure 2G**), and compact (**Figure 2H**) patterns.

### 2.5 Clinical data processing and correlation analysis of HCC patients

We used the Barcelona Clinic Liver Cancer (BCLC) staging system (36), widely employed internationally for treatment guidance, as a reference. We focused on BCLC stage 0, stage A, and the first subgroup of stage B, where patients in the first subgroup of stage B underwent liver resection due to the lack of liver donors. Additionally, we performed univariate and multivariate Cox regression analyses based on OS and plotted Kaplan-Meier curves. Finally, to investigate the impact of clinical and pathological variables on Tim-3 expression, we conducted univariate logistic regression analysis on all variables (**Table 1**), and included variables with p-values less than 0.05 in the multivariate analysis model (**Table 2**).

**Table 1:** Correlation between clinical and pathological characteristics and Tim-3 expression.

Characteristics	Low Expression (n=52)	High Expression (n=50)	Z/x2	P-value
Gender			2.152	0.142
Male	40	44		
Female	12	6		
Age	54.810 **±** 14.059	58.740 **±** 13.638	-1.433	0.155*
BMI	23.204 **±** 3.251	22.388 **±** 3.284	1.261	0.210*
Family history				0.161
Negative	45	48		
Positive	7	2		
Viral hepatitis			0.314	0.575
Negative	17	19		
Positive	35	31		
Cirrhosis			0.810	0.368
Negative	12	8		
Positive	40	42		
Tumor number			0.005	0.942
1	46	44		
≤3	6	6		
Tumor size			3.175	0.075
<5	36	26		
≥5	16	24		
AFP (ng/mL)			0.118	0.731
<200	39	36		
≥200	13	14		
Child-Pugh score			2.761	0.097
A	41	32		
B	11	18		
BCLC score			-2.352	0.019
0	24	10		
A	23	35		
B	5	5		
TNM stage(I/II,III)			0.942	0.332
I	34	28		
II,III	18	22		
Pathological grading			1.095	0.613
Well	2	4		
Moderately	47	42		
Poorly	3	4		
MVI			0.343	0.558
Negative	42	38		
Positive	10	12		
Satellite nodules			0.211	0.646
Negative	43	43		
Positive	9	7		
Histological morphology			35.751	0.000
Trabecular-micro	36	11		
Trabecular-macro	4	20		
Pseudoglandular	10	5		
Compact types	2	14		
ALT	29.850(21.325-46.050)	41.950(29.308-58.568)	-1.951	0.051
AST	30.150(24.575-40.125)	44.700(27.575-72.950)	-2.775	0.006

Data marked with an asterisk (*) were analyzed using the student’s T-test.

**Table 2:** Univariate and multifactorial regression of Tim-3 with clinicopathological data.

Characteristics	Univariate regression		multifactorial regression	
	OR (95% CI)	P-value	OR (95% CI)	P-value
Gender	0.455(0.156-1.324)	0.148		
Age	1.021(0.992-1.051)	0.156		
BMI	0.925(0.820-1.044)	0.209		
Family history	0.268(0.053-1.358)	0.112		
Viral hepatitis	1.262(0.559-2.847)	0.575		
Cirrhosis	1.575(0.583-4.255)	0.370		
Tumor number	1.045(0.313-3.487)	0.942		
Tumor size	2.077(0.925-4.664)	0.077		
AFP (ng/mL)	1.161(0.499-2.701)	0.729		
Child-Pugh score	2.097(0.869-5.060)	0.100		
BCLC score	2.083(1.064-4.075)	0.032	2.240(1.065-4.712)	0.033
TNM Stage(I/IIIII)	1.484(0.668-3.299)	0.333		
Pathological grading	0.860(0.289-2.559)	0.786		
MVI	1.326(0.514-3.419)	0.559		
Satellite nodules	0.778(0.266-2.277)	0.647		
Histological morphology	2.183(1.442-3.304)	<0.001	2.241(1.456-3.448)	<0.001
ALT	1.003(0.997-1.008)	0.321		
AST	1.005(0.998-1.013)	0.163		

### 2.6 Image acquisition

All participants underwent CECT scans (SOMATOM Definition AS 128-slice spiral CT scanner) within 4 weeks before surgery. The scanning parameters were as follows: tube voltage 120KV, tube current 250mA, slice interval 5mm, slice thickness 5.0mm, rotation speed 0.6s, pitch 0.5, collimator 16×0.625mm, and matrix 512×512. The scans were performed in the supine position, covering from the glabella to the symphysis pubis. The arterial phase began 30-35 seconds after injection, the venous phase began 55-60 seconds after injection, and the delayed phase began 90-120 seconds after injection. Images were reconstructed in coronal, sagittal, and other planes using MultiPlanar Reconstruction technology and then uploaded to the Picture Archiving and Communication System for storage.

### 2.7 Region of interest segmentation and image preprocessing

A radiologist with over 5 years of experience used ITK-SNAP 3.8.0 to manually delineate regions of interest (ROI) layer by layer on the arterial phase enhancement (ACE) and portal venous phase enhancement (VCE) CECT images (**Figure 1B**). The radiologist was blinded to the patients’ clinical information and pathological results. Pixel values were normalized to the range of -200 to 300 to mitigate the impact of extreme pixel values. Resampling to a fixed resolution of 1mm×1mm×1mm was performed to achieve consistent voxel spacing.

### 2.8 Traditional radiomics model and habitat-based radiomics model

#### 2.8.1 Habitat generation

**Figure 1B** illustrates the complete workflow of our habitat analysis. First, we used the Simple Linear Iterative Clustering method to segment the superpixels within the ROI. Then, we applied the K-means unsupervised clustering method to divide the ROI of each sample into three distinct cluster centers, each corresponding to a habitat region (37). For each subregion, we extracted shape, texture, and first-order features. Additionally, to minimize the inefficacy of radiomics feature extraction in subregions, we balanced color and spatial proximity and filtered out smaller subregions.

#### 2.8.2 Feature extraction

The handcrafted features in this study were categorized into geometric, intensity, and texture features. Geometric features depicted the three-dimensional shape of the tumor. Intensity features provided statistical analysis of voxel intensities. Various methods were employed to extract these texture features, including Gray Level Co-occurrence Matrix (GLCM), Gray Level Run Length Matrix (GLRLM), Gray Level Size Zone Matrix (GLSZM), and Neighboring Gray Tone Difference Matrix (NGTDM). For habitat analysis, specific features for each identified subregion were extracted.

Since the clustering algorithm we used is unsupervised, the labels for each subregion after clustering are not guaranteed to be consistent. To ensure that different habitat regions maintained the same or similar physical significance, we used the ReMap technique to redraw similar regions, detailed in **Supplementary Figure 1A**. All features were extracted using the pyradiomics library (http://pyradiomics.readthedocs.io), with most features following the definitions specified by the Imaging Biomarker Standardization Initiative (IBSI).In our experiments, we extracted features at two different phases: arterial phase enhancement ACE and VCE. Subsequently, the extracted features were combined using an early fusion method to obtain a unified feature set. This integrated feature set effectively represents the characteristics of the habitat regions across both modalities.

#### 2.8.3 Feature selection

To assess the robustness of image features, we performed retest analysis and inter-rater reliability assessment: the retest analysis involved the original rater re-segmenting the ROI for a randomly selected group of 30 patients, while the inter-rater reliability assessment involved two raters independently segmenting the ROI subregions for another randomly selected group of 30 patients. The ICC was used to evaluate the features extracted from the subregions, with features having an ICC ≥ 0.85 considered robust against segmentation uncertainty.

Features fitting a t-distribution were selected, and highly reproducible features were further analyzed using Pearson correlation coefficients to identify strong correlations. The optimal λ value for the Lasso (Least Absolute Shrinkage and Selection Operator) regression model was determined using 10-fold cross-validation, selecting the value corresponding to the lowest mean standard error (**Supplementary Figures 1B–E**).

### 2.9 Radiomics labels

In this study, we focused on evaluating the effectiveness of different tumor region analyses in predicting Tim-3 expression. These analyses included two approaches: considering the tumor region as a whole (Rad) and analyzing specific tumor habitats (Habitat). When developing the Rad risk model, we treated the ROI as a single entity and extracted radiomic features. The development of the Habitat model was based on the K-means unsupervised clustering algorithm. Due to this unsupervised approach, clusters with the same centers might not convey the same physical meaning. To address this challenge, we averaged these features.

The feature selection process for habitat signatures differs from standard methods as it excludes the ICC assessment. In developing Rad and Habitat risk models, we employed widely recognized machine learning models, including Logistic Regression (LR) for linear models, Random Forest, Extra Trees, XGBoost, and LightGBM. We used the receiver operating characteristic (ROC) curve in the test cohort to compare and evaluate each model. Additionally, calibration curves were generated to assess the accuracy of model calibration, supplemented by the Hosmer-Lemeshow goodness-of-fit test to further evaluate calibration capability. Decision curve analysis (DCA) was also employed to evaluate the clinical utility of the models. Finally, to better mitigate the potential impact of insufficient sample size in the test set, we used 5-fold cross-validation with features selected by the Rad and Habitat models to test model stability.

### 2.10 Statistical analysis

Comparisons between categorical variables were performed using the χ² or Fisher’s Exact test. Comparisons between continuous variables that were normally distributed and met the homogeneity of variance were performed using the t-test, while comparisons between continuous variables that were not normally distributed were performed using the Mann-Whitney U test. A two-sided p-value < 0.05 was considered statistically significant. Data analysis was conducted using IBM SPSS Statistics (v.26.0; IBM Corporation, Armonk, NY). Survival analysis was estimated using Kaplan-Meier curves, and OS was evaluated using the log-rank test. Additionally, data visualization and some statistical analyses were performed using R (version 4.0.0; R Foundation for Statistical Computing, Vienna, Austria). Specifically, the “survival” package was used for Cox regression analysis and Kaplan-Meier curve plotting, and the “ggplot2” package was used for data visualization.

## 3 Results

### 3.1 Analysis of Tim-3 expression and prognosis based on the TCGA database

**Figure 3:**
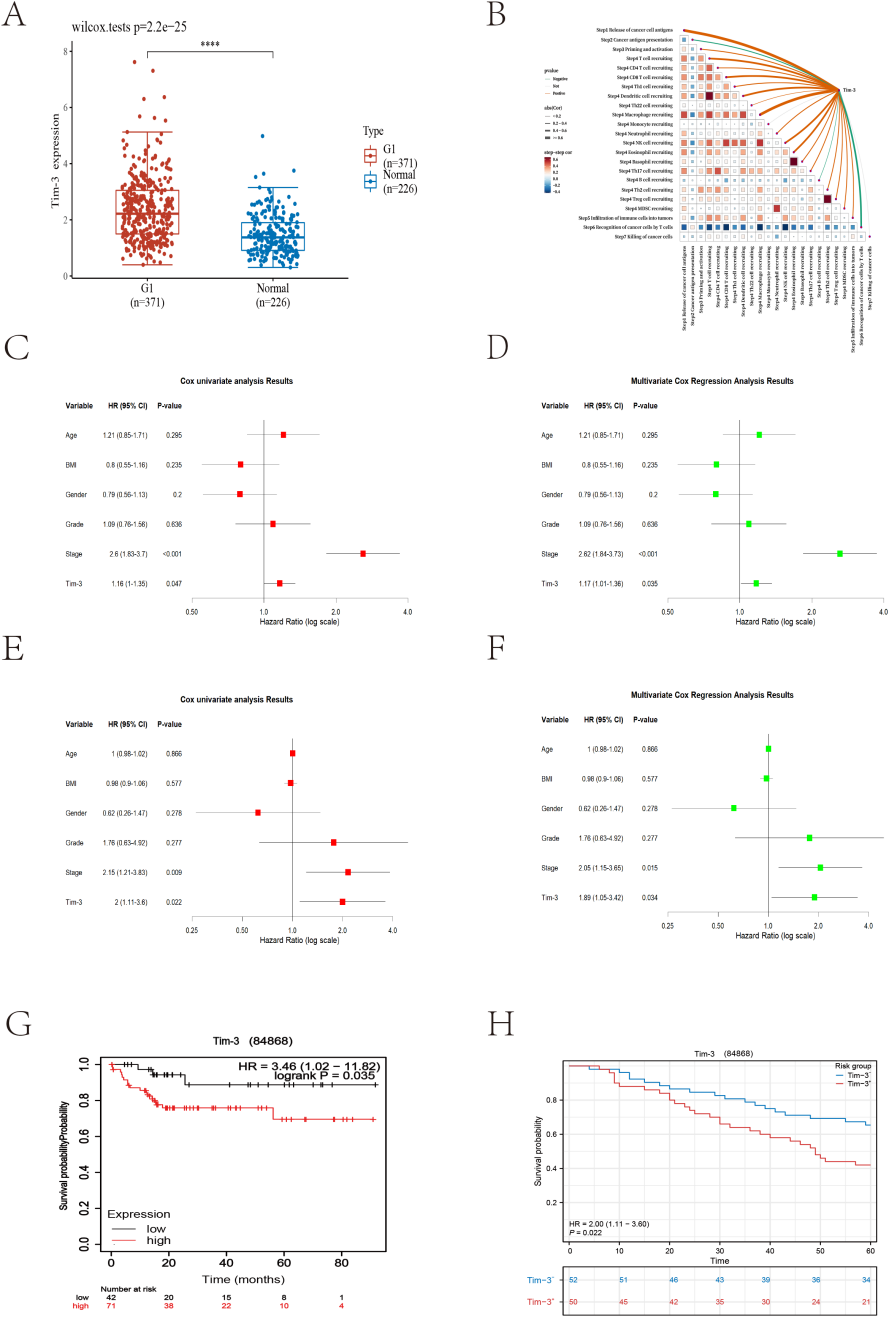
**(A)** Differences in Tim-3 expression between tumor cells and normal liver tissue (p<0.001). **(B)** Bioinformatics analysis reveals the relationship between Tim-3 and the liver cancer immune cycle. **(C)** Univariate Cox regression analysis of bioinformatics datasets. **(D)** Multivariate Cox regression analysis of bioinformatics datasets. **(E)** Univariate Cox regression analysis of local datasets. **(F)** Multivariate Cox regression analysis of local datasets. **(G, H)** Kaplan-Meier curves for overall survival (OS) from bioinformatics and local datasets.

The analysis results showed that Tim-3 was significantly overexpressed in HCC tissues compared to normal tissues (**Figure 3A**). Using the TIP database to analyze the scores of various cancer immune cycle steps, it was found that the Tim-3 gene performed better in step 1 (cancer cell antigen release) and step 4 (immune cell transport to the tumor) (**Figure 3B**). Univariate Cox regression analysis showed that Tim-3 (p=0.047) and stage (p<0.001) were significant risk factors (**Figure 3C**). Subsequent multivariate Cox regression analysis included Tim-3 (p=0.035) and stage (p<0.001) in the model (**Figure 3D**). The Kaplan-Meier curve showed that high expression of Tim-3 in the Asian population was significantly associated with poorer overall survival (OS) in patients with early resectable HCC (p=0.035) (**Figure 3G**).

### 3.2 Analysis of Tim-3 expression and prognosis based on single-center data

As of December 2023, 104 cases had reached the five-year follow-up endpoint. One case was excluded due to the lack of tumor cells in the IHC-P results, and another was omitted during model fitting. Among the remaining 102 cases, 61 patients experienced recurrence, and 47 patients died of HCC during the follow-up period. The recurrence rates at one, three, and five years were 29.4%, 51.0%, and 59.8%, respectively; the overall survival rates at one, three, and five years were 90.1%, 70.6%, and 53.9%, respectively. Consistent with the trend of the external validation results, univariate Cox regression analysis showed that Tim-3 (p=0.022) and stage (p=0.009) were significant risk factors (**Figure 3E**). Subsequent multivariate Cox regression analysis included Tim-3 (p=0.034) and stage (p=0.015) in the model (**Figure 3F**).

### 3.3 Correlation analysis between Tim-3 expression and clinicopathological characteristics

Using X-tile to determine the optimal cutoff value based on patients’ OS, we divided the patients into high Tim-3 (≥92.3) and low Tim-3 (<92.3) groups. We examined the correlation between Tim-3 immunohistochemical variables and clinicopathological characteristics. The results showed that Tim-3 was correlated with patients’ BCLC stage (p=0.019) and AST levels (p=0.006), but not with other clinical characteristics. Additionally, high Tim-3 expression was associated with the macrotrabecular-massive and compact subtypes (p<0.001), but not with microvascular invasion, satellite nodules, or other factors (p>0.05) (**Table 1**). Univariate and multivariate analyses indicated that BCLC stage (p=0.033), macrotrabecular-massive, and compact subtypes (p<0.001) were independent risk factors for high Tim-3 expression (**Table 2**). Kaplan-Meier survival analysis revealed that high Tim-3 expression was associated with poorer OS (p=0.022) (**Figure 3H**).

### 3.4 Feature statistics

**Figure 4:**
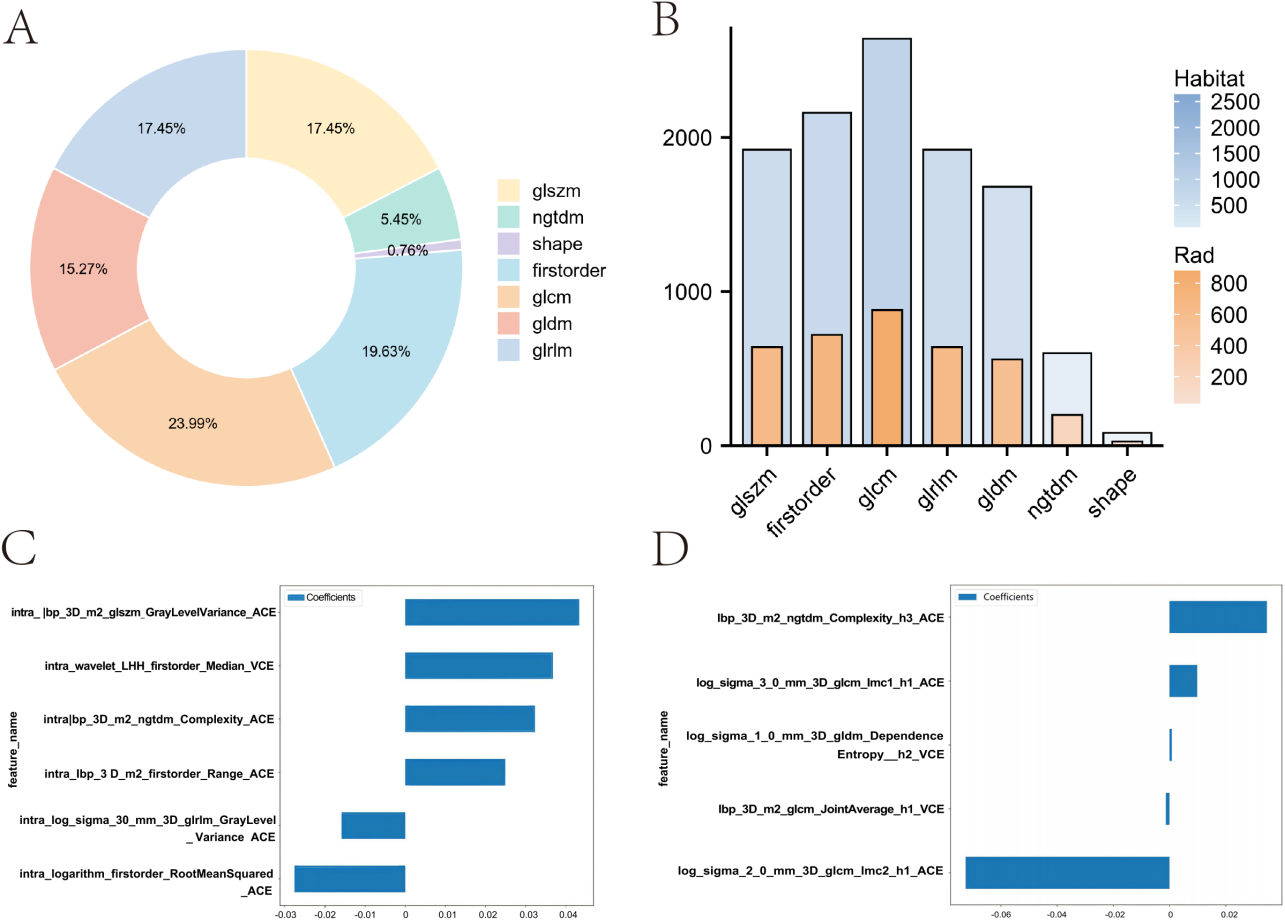
Comparison of feature distribution and weights between Rad and Habitat models. **(A)** Doughnut plot showing the proportion of local features. **(B)** Bar chart comparing the number of features in Rad and Habitat models. **(C)** Feature weights in the Rad model. **(D)** Feature weights in the Habitat model.

In this study, for the Rad model, we extracted 1,834 handcrafted radiomic features from each case’s arterial and venous phases. In contrast, for the Habitat model, we extracted features from three regions in the arterial and venous phases, resulting in a total of 11,004 features per patient. These features include 360 first-order features, 14 shape-based features, and various texture features such as the gray-level co-occurrence matrix (GLCM), gray-level run length matrix (GLRLM), gray-level size zone matrix (GLSZM), and neighboring gray-tone difference matrix (NGTDM). The proportions of the extracted features are shown in **Figure 4A**, and the comparison of feature counts between the Rad and Habitat models is depicted in **Figure 4B**.

### 3.5 Lasso-based radiomics feature selection

We implemented a multitask Lasso CV method, which is suitable for multitask learning in a single model, to accommodate our data. This method, combined with 10-fold cross-validation, was used for the selection of Rad features and Habitat features (**Supplementary Figures 1B-E**). In the Rad model, a total of six imaging features were incorporated, with GLSZM contributing the most (**Figure 4C**). In contrast, the Habitat model incorporated five imaging features, including three arterial-phase features and two venous-phase features, with the arterial-phase features from habitat regions 1 and 3 contributing most to model fitting. These features include GLCM and NGTDM (**Figure 4D**).

### 3.6 Radiomics model fitting

**Figure 5:**
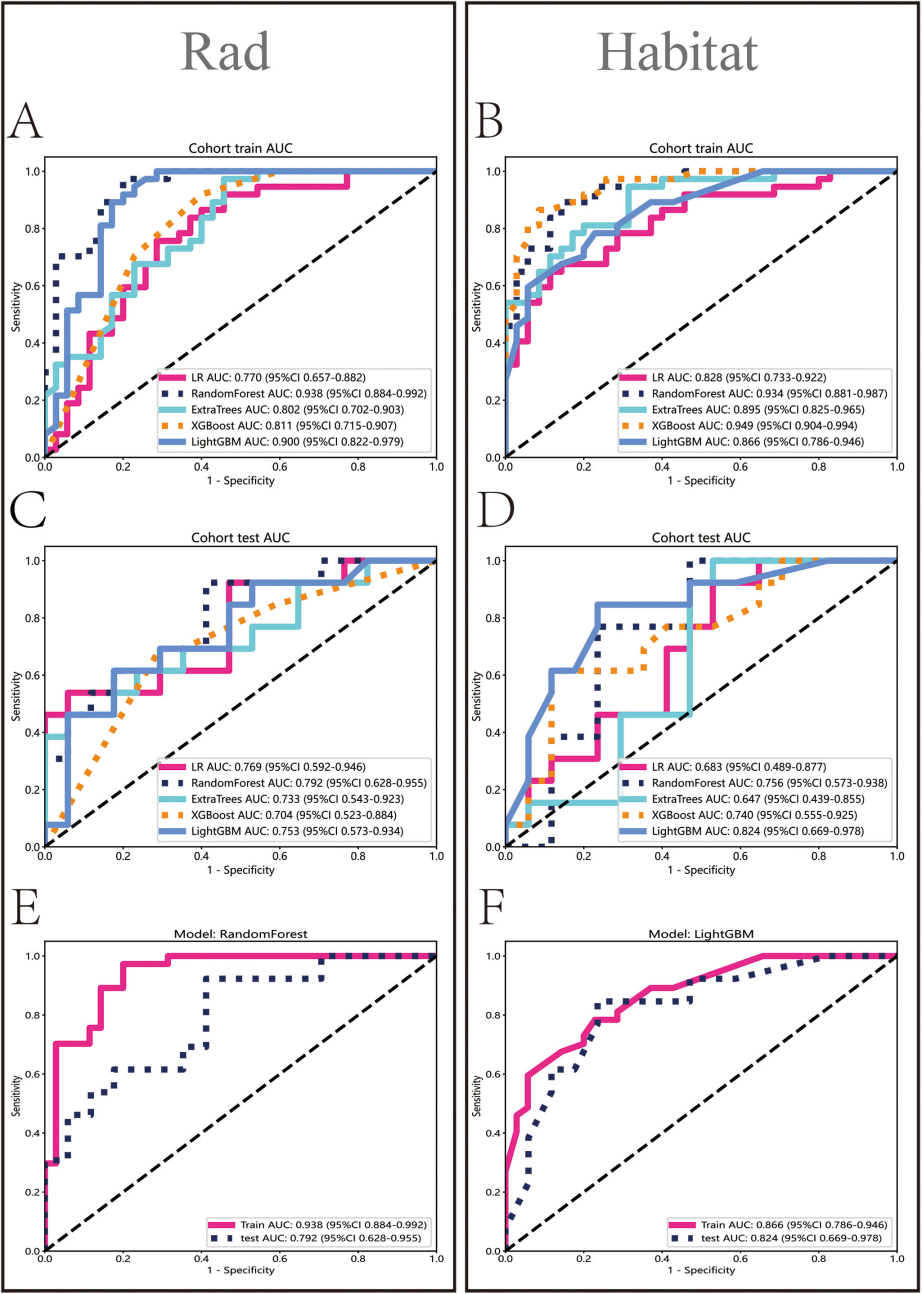
Performance evaluation of Rad and Habitat models. The figure illustrates the performance of Rad and Habitat models using algorithms such as LR, RandomForest, ExtraTrees, XGBoost, and LightGBM on the training dataset **(A, B)** and testing dataset **(C, D)**. **(E, F)** specifically highlight the performance of the most effective algorithms, which are RandomForest (Rad) and LightGBM (Habitat), respectively.

**Figure 6:**
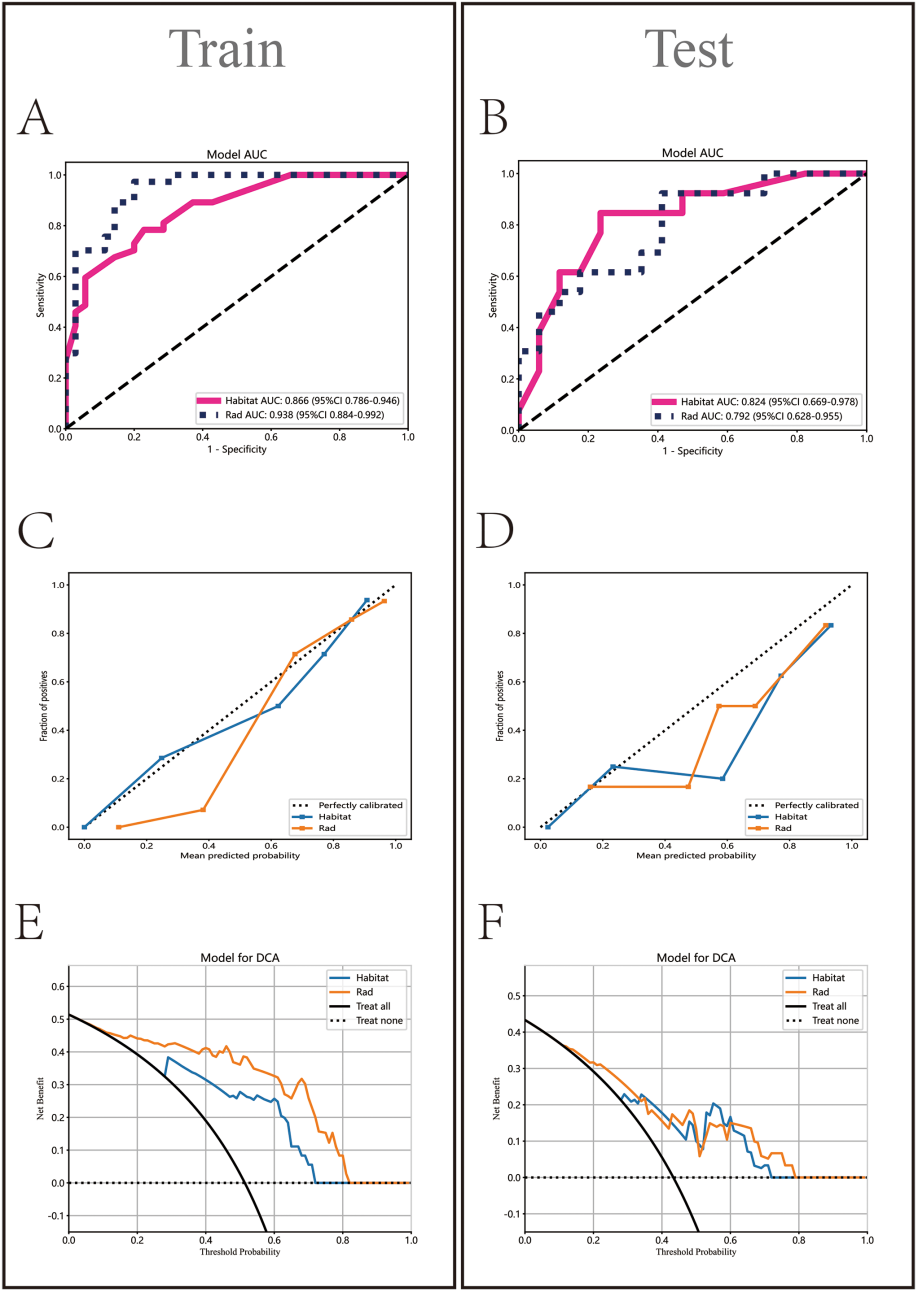
Comparative analysis of Rad and Habitat models on training and testing datasets. It sequentially displays the ROC curves (AUC values) **(A, B)**, calibration curves **(C, D)**, and decision curves **(E, F)** for both models.

In the Rad model, the AUC values generated by five methods for the training set ranged from 0.770 to 0.938 (**Figure 5A**), while in the Habitat model, the training set AUC values ranged from 0.828 to 0.949 (**Figure 5B**). In the test set, the Rad model’s AUC values ranged from 0.704 to 0.792 (**Figure 5C**), and the Habitat model’s AUC values ranged from 0.647 to 0.824 (**Figure 5D**). Based on the best AUC values, we selected the Random Forest algorithm for the Rad model, achieving AUCs of 0.938 in the training set and 0.792 in the test set (**Figure 5E**). For the Habitat model, we selected the Light GBM algorithm, achieving AUCs of 0.866 in the training set and 0.824 in the test set (**Figure 5F**). A comparison of the models revealed that while the Rad model (**Figure 6A**) demonstrated a higher AUC in the training set (Rad vs. Habitat: 0.928 vs. 0.866), its performance in the test set (Rad vs. Habitat: 0.792 vs. 0.826) was inferior to that of the Habitat model (**Figure 6B**).

### 3.7 Model performance evaluation and comparison

**Figure 7:**
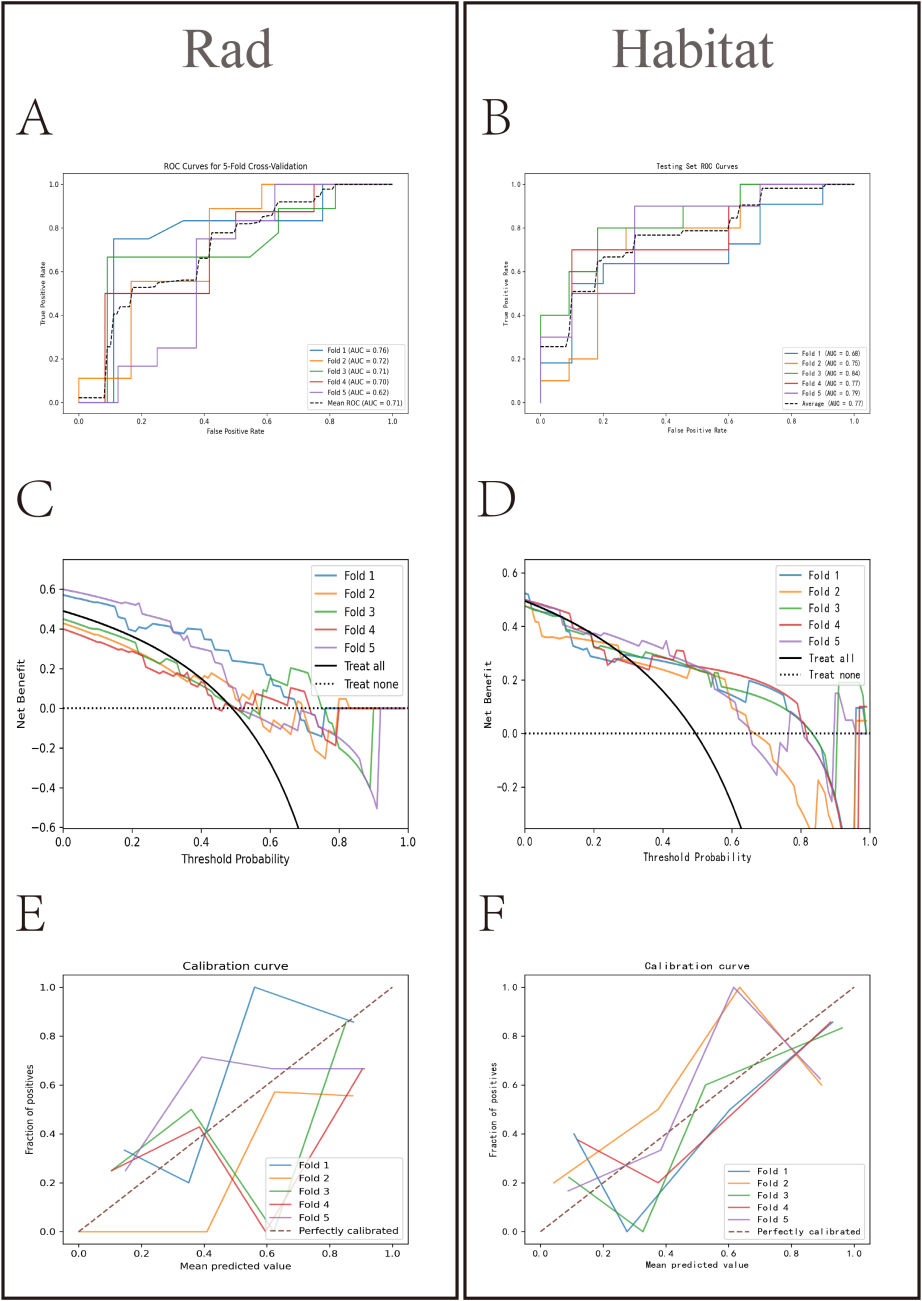
Comparative analysis of Rad and Habitat models in five-fold cross-validation. The left panel showing the performance of the Rad model on the RandomForest testing dataset for each fold, and the right panel showing the performance of the Habitat model on the LightGBM testing dataset for each fold. It sequentially presents the ROC curves (AUC values) **(A, B)** decision curves **(C, D)** and calibration curves **(E, F)** for both models.

HL test (**Figures 6C, D**) showed that the Habitat model exhibited the highest calibration performance. Decision curve analysis evaluated the performance of these models (**Figures 6E, F**), and the results indicated that the Habitat model showed higher net benefits across most threshold ranges. We used five-fold cross-validation to validate both models, with the average AUCs in the validation set being Rad (0.71) and Habitat (0.77) (**Figures 7A, B**). This suggests that compared to the Rad model, the Habitat model has superior decision-making performance in predicting Tim-3 expression status, especially within clinically relevant threshold ranges. The DCA curves from five-fold cross-validation demonstrate that the Rad model (**Figure 7C**) offers less clinical benefit compared to the Habitat model (**Figure 7D**). Further validation analysis revealed that the Rad model (**Figure 7E**) was not well-calibrated, whereas the Habitat (**Figure 7F**) model demonstrated better performance.

## 4 Discussion

Radiogenomics is an important method for predicting immunotherapy biomarkers. In our study, we first identified the potential of Tim-3 as a prognostic biomarker for HCC. Subsequently, we constructed two classification models (Rad and Habitat) based on radiomic features from CECT. We developed an HCC Tim-3 expression model using the better-performing Habitat model.

Our study showed that Tim-3 is significantly elevated in HCC tissues compared to normal liver tissues (p<0.001). COX regression analysis indicated that high Tim-3 expression and tumor stage are independent risk factors for OS in HCC patients. This result is supported by several studies (12, 16). We found no significant difference in TNM stage between the high Tim-3 expression group and the low Tim-3 expression group, which contrasts with the findings of Li et al. (15). This discrepancy may be due to Li et al.’s focus on HBV-HCC, suggesting potential selection bias in our study. Analysis of Tim-3 expression status revealed that Tim-3 is expressed in a significant number of intrinsic tumor cells and tumor stroma in HCC. The liver cancer immune cycle results also show that Tim-3 performs better in the steps of cancer cell antigen release and immune cell tumor chemotaxis. This indicates that Tim-3 may play a role in promoting liver cancer cell antigen release and affecting the function of T cells, NK cells, macrophages, and dendritic cells. This suggests that Tim-3 may promote tumor progression by affecting both intrinsic liver cancer cells (38) and immune cell function (39, 40) in multiple ways. Anti-Tim-3 is becoming an important target for cancer immunotherapy. Studies have shown that the use of nanoparticles to enhance the co-delivery of Tim-3 siRNA and sorafenib can improve anti-HCC effects (18). Zhang et al. (38) found that anti-Tim-3 antibodies can reverse the tumor-promoting effects of endogenous Tim-3 in hepatocytes. Combined with previous liver cancer immune cycle results, this evidence demonstrates that anti-Tim-3 therapy may achieve stronger anti-tumor effects by acting on both intrinsic malignant cells and immune cells, further supporting its potential as a targeted therapeutic marker.

In this study, we analyzed ACE and VCE images from preoperative enhanced CT scans of HCC patients and constructed two models (Rad and Habitat) to predict Tim-3 expression. The AUCs in the training and test sets were (0.938 vs 0.866) and (0.792 vs 0.824), respectively. We found that using habitat radiomics to predict the expression of the immune checkpoint Tim-3 in HCC showed a more significant advantage compared to traditional radiomics. This may be due to traditional radiomics focusing on analyzing the entire tumor or peritumoral area as a whole (41), whereas habitat analysis emphasizes subregions with different metabolic characteristics, providing a better explanation for Tim-3’s impact on the tumor TME (27).

Compared to general machine learning studies (42, 43), our models implemented multiple measures to ensure feature extraction stability and avoid overfitting. First, we used repeatability analysis and inter-rater reliability assessments to select stable features. Second, we compared the performance of models fitted using methods such as Random Forest, Logistic Regression, Extra Trees, Light GBM, and XG Boost, selecting the Light GBM with the best AUC to build the Habitat model. During model fitting, the GLCM and NGTDM contributed the most, both extracted from arterial phase images, describing image texture features based on pixel contrast. Gong et al. (25) also demonstrated that the main features in fitting PD-1/PD-L1 models came from GLCM, suggesting that pixel contrast features may aid in TME analysis and further help stratify HCC patients for immunotherapy (26). Finally, we comprehensively evaluated model performance using DCA and calibration curves, and used five-fold cross-validation to mitigate potential impacts from insufficient data volume. The results indicate that our models performed well in assessing Tim-3 expression status, potentially aiding clinicians in making timely clinical decisions.

This study has certain limitations. First, although we used a non-invasive method to attempt predicting Tim-3 expression and prognosis in resectable HCC patients, the sample size was small, limiting the generalizability of the results. Second, the findings have not yet been validated in multi-center studies, highlighting the necessity for further validation. Additionally, we lack prospective studies to evaluate the effectiveness of radiomics in predicting prognosis after neoadjuvant therapy. Therefore, more studies are needed to verify and expand upon these preliminary findings.

In conclusion, the habitat radiomics model based on CECT has the potential to predict Tim-3 expression status in HCC and could serve as a biomarker for Tim-3 targeted therapy.

## Data Availability

The raw data supporting the conclusions of this article will be made available by the authors, without undue reservation.

## Supplementary material

Supplementary Figure 1
**(A)** ReMap process. **(B)** Lasso regression of the Rad model. **(C)** Lasso regression of the Habitat model. **(D)** Mean Squared Error (MSE) of the Rad model. **(E)** Mean Squared Error (MSE) of the Habitat model.
