# Supplementary figures and images for: Unveiling Tim-3 immune checkpoint expression in hepatocellular carcinoma through abdominal contrast-enhanced CT habitat radiomics
Source: Front Oncol. 2024 Nov 8;14:1456748. doi: 10.3389/fonc.2024.1456748 (PMC11581969; doi:10.3389/fonc.2024.1456748)

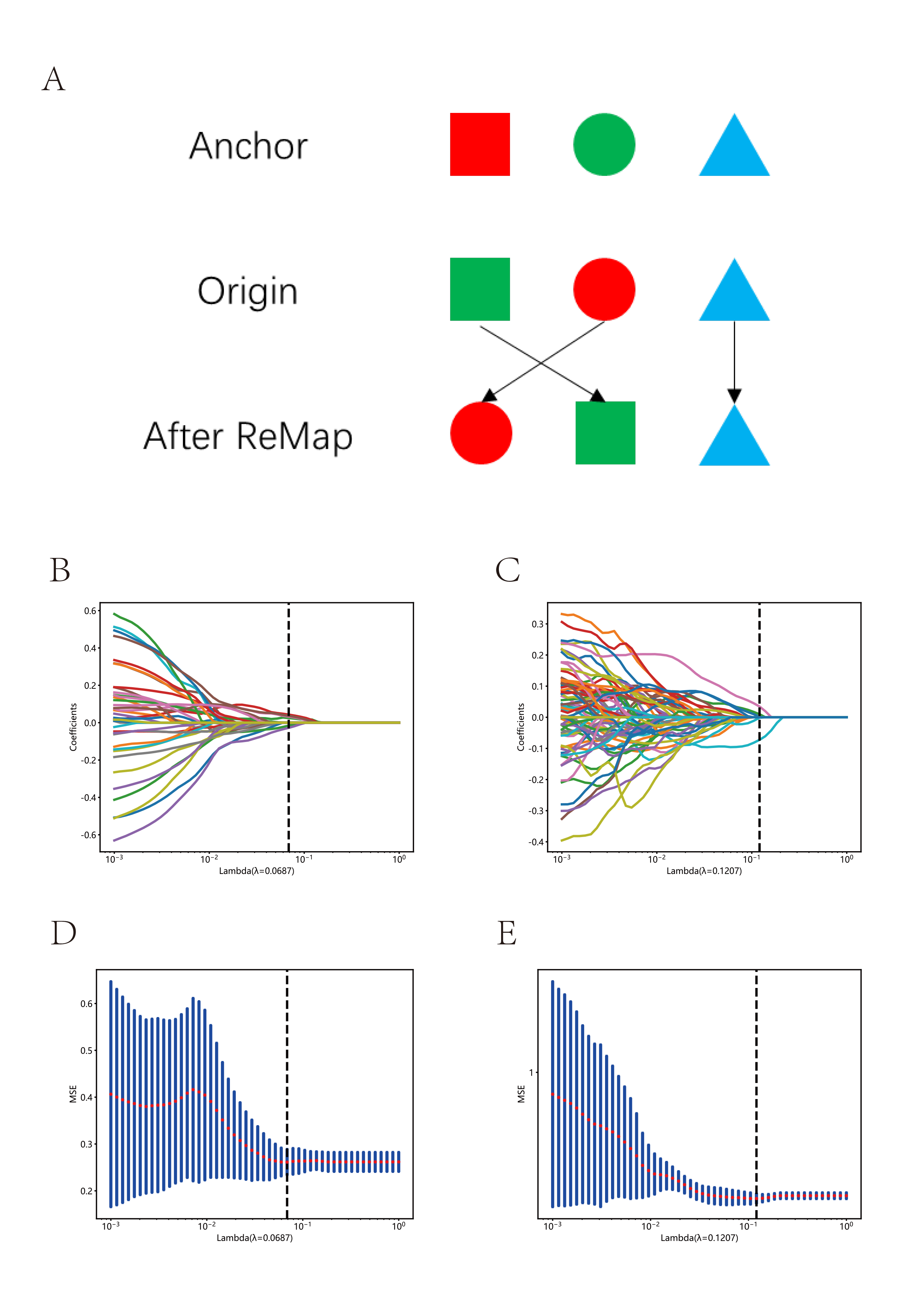

Supplement: Supplementary Figure 1 — (A) ReMap process. (B) Lasso regression of the Rad model. (C) Lasso regression of the Habitat model. (D) Mean Squared Error (MSE) of the Rad model. (E) Mean Squared Error (MSE) of the Habitat model. [file Image1.tif]
