# Supplementary material for: Unveiling Tim-3 immune checkpoint expression in hepatocellular carcinoma through abdominal contrast-enhanced CT habitat radiomics
Source: Front Oncol. 2024 Nov 8;14:1456748. doi: 10.3389/fonc.2024.1456748 (PMC11581969; doi:10.3389/fonc.2024.1456748)
Supplement: Supplementary file 2 [file DataSheet1.docx]

## Supplementary

### **1A: Description of the Remap Algorithm**

In a dataset, assume each sample consists of $n$ feature vectors. An anchor point is established, which can be predefined or selected based on certain characteristics of the dataset. For each sample in the dataset, calculate the distance between a specific feature vector and the anchor point. Based on this distance, reorganize all feature vectors for each sample, arranging them so that vectors closer to the anchor point are prioritized in the order.

The objective of this process is to restructure each sample's feature vectors to better reflect their relational strength with the anchor point. This approach can be particularly beneficial in feature selection, dimensionality reduction, or data preprocessing, especially in scenarios where emphasizing relevance to a specific reference point is crucial.

Step 1: Define the Dataset and Anchor Point

- Assume a dataset $D$ where each sample $s_{i}$ (for $i=1,2,...,m$) consists of $n$ feature vectors.
- Define an anchor point $A$. This anchor can be predefined or selected based on certain characteristics of the dataset.

Step 2: Calculate Distance to the Anchor Point

- For each sample $s_{i}$ in the dataset, calculate the distance between a specific feature vector $v_{ij}$ (where $j$ is a specific feature dimension) and the anchor point $A$.
- Use a distance function $d\left( v_{ij},A \right)$ to compute this.
- For each feature vector in sample $s_{i}$, calculate $d_{ij}=d\left( v_{ij},A \right)$.

Step 3: Sort Feature Vectors Based on Distance

- Sort the feature vectors in each sample $s_{i}$ based on the calculated distances $d_{ij}$.
- The sorting function $\sigma$ such that $\sigma\left( s_{i},A \right)=sort\left( s_{i},byd_{ij} \right)$ will reorder the feature vectors.
- The reordered sample is represented as $s'_{i}=v'_{i1},v'_{i2},...,v'_{in}$, where $v'_{ij}$ are the feature vectors reordered based on their distances $d_{ij}$ from the anchor point.

Step 4: Form the Final Dataset

- Apply the above steps to all samples in the dataset.
- The final dataset $D'$ will consist of all the reordered samples $s'_{i}$ (for $i=1,2,...,m$).

### 2: **Description of Local Features**

Local features in a dataset provide detailed insights into specific aspects or characteristics of the data. These features could include various measurements or attributes of the data points, such as intensity, texture, or other statistical properties. In many cases, local features help in understanding the intricate details of the dataset, enabling more precise modeling or analysis.

A total of 19 local features were extracted in this study, specifically: firstorder_Entropy, firstorder_MeanAbsoluteDeviation, firstorder_Median, glcm_DifferenceAverage, glcm_DifferenceEntropy, glcm_DifferenceVariance, glcm_Imc1, glcm_Imc2, glcm_InverseVariance, glcm_JointEnergy, glcm_JointEntropy, glcm_SumEntropy, glrlm_LongRunEmphasis, glrlm_RunEntropy, glrlm_RunVariance, glszm_SizeZoneNonUniformityNormalized, glszm_SmallAreaHighGrayLevelEmphasis, ngtdm_Contrast, ngtdm_Strength. The definitions for these typical features are:

**Entropy**: Entropy specifies the uncertainty/randomness in the image values.

$$entropy=-\sum_{i=1}^{N_{g}} p\left( i \right)\log_{2}\left( p\left( i \right)+\epsilon\right)$$

**Mean Absolute Deviation (MAD)** : MAD is the mean distance of all intensity values from the Mean Value of the image array.

$$MAD=\frac{1}{N_{p}}\sum_{i=1}^{N_{p}} \left| X\left( i \right)-X \right|$$

**Difference Entropy**: Measures the randomness/variability in neighborhood intensity value differences.

$$difference\_entropy=\sum_{k=0}^{N_{g}-1} p_{x-y}\left( k \right)\log_{2}\left( p_{x-y}\left( k \right)+\epsilon\right)$$

**Difference Variance**: A measure of heterogeneity, giving higher weights to differing intensity level pairs.

$$difference\_variance=\sum_{k=0}^{N_{g}-1} \left( k-DA \right)^{2}p_{x-y}\left( k \right)$$

**Joint Energy**: A measure of homogeneous patterns in the image.

$$joint\_energy=\sum_{i=1}^{N_{g}} \sum_{j=1}^{N_{g}} \left( p\left( i,j \right) \right)^{2}$$

**Joint Entropy**: Measures the randomness/variability in neighborhood intensity values.

$$joint\_entropy=-\sum_{i=1}^{N_{g}} \sum_{j=1}^{N_{g}} p\left( i,j \right)\log_{2}\left( p\left( i,j \right)+\epsilon\right)$$
